# Emetine, Ipecac, Ipecac Alkaloids and Analogues as Potential Antiviral Agents for Coronaviruses

**DOI:** 10.3390/ph13030051

**Published:** 2020-03-21

**Authors:** Martin D. Bleasel, Gregory M. Peterson

**Affiliations:** School of Pharmacy and Pharmacology, University of Tasmania, Hobart Tasmania 7001, Australia; martin.bleasel@gmail.com

**Keywords:** COVID-19, coronavirus, emetine, ipecac, dehydroemetine, MERS, SARS, treatment, repurposing: antiviral

## Abstract

The COVID-19 coronavirus is currently spreading around the globe with limited treatment options available. This article presents the rationale for potentially using old drugs (emetine, other ipecac alkaloids or analogues) that have been used to treat amoebiasis in the treatment of COVID-19. Emetine had amongst the lowest reported half-maximal effective concentration (EC_50_) from over 290 agents screened for the Middle East respiratory syndrome (MERS) and severe acute respiratory syndrome (SARS) coronaviruses. While EC_50_ concentrations of emetine are achievable in the blood, studies show that concentrations of emetine can be almost 300 times higher in the lungs. Furthermore, based on the relative EC_50_s of emetine towards the coronaviruses compared with *Entamoeba histolytica*, emetine could be much more effective as an anti-coronavirus agent than it is against amoebiasis. This paper also discusses the known side effects of emetine and related compounds, how those side effects can be managed, and the optimal method of administration for the potential treatment of COVID-19. Given the serious and immediate threat that the COVID-19 coronavirus poses, our long history with emetine and the likely ability of emetine to reach therapeutic concentrations within the lungs, ipecac, emetine, and other analogues should be considered as potential treatment options, especially if in vitro studies confirm viral sensitivity.

Emetine is one of the main alkaloids found in ipecacuanha (ipecac) root [1]. Ipecac syrup has predominantly been used to induce vomiting in the management of poisoning. Emetine, and perhaps ipecac, ipecac alkaloids and their analogues, should be considered as potentially potent and effective therapeutic agents against the coronavirus family of viruses and, in particular, the COVID-19 coronavirus. As an antiviral, emetine has been shown to have amongst the lowest EC_50_ value (half-maximal effective concentration) from over 294 agents screened, with an EC_50_ of 0.054 and 0.014 μM for the severe acute respiratory syndrome (SARS) and the Middle East respiratory syndrome (MERS) coronaviruses, respectively [2,3]. Outlined here is the rationale for the potential use of emetine or similar compounds in the treatment of coronavirus infections and, if confirmed from in vitro sensitivity analyses, COVID-19.

Emetine, as an isolated alkaloid, was used as an anti-infective agent from 1912 when Vedder [4] showed that the drug killed amoebae in vitro. It was one of the most widely used agents, orally or intramuscularly, in the treatment of both intestinal and extraintestinal amoebiasis [5,6,7,8] until metronidazole became available [5]. Emetine is structurally unrelated to metronidazole.

In a study looking at the amount of emetine absorbed after a 30 mL oral dose of ipecac (containing 13.9 mg of emetine) in 10 subjects, the mean plasma concentration between 1 and 3 h post-dosing was approximately 6.5 ng/mL (0.0135 μM), which equates to only 0.25 to ~1 times the EC_50_ for SARS and MERS coronaviruses, with all patients vomiting at the 30 mL dose [9]. While the emetic effects of ipecac syrup (30 mL) can be completely eliminated and nausea significantly reduced by the use of 5-HT3 antagonists, such as ondansetron [10], which may enable higher blood levels to be achieved, these results might initially appear uninspiring. However, as coronaviruses are predominantly respiratory tract infections, the amount of emetine in the lungs is more important than in the plasma. Oral bioavailability studies of ipecac alkaloids in rats have shown that the concentration of radiolabeled emetine is many times higher in the tissues than in plasma. In relation to the lungs, after 8 and 24 h, the concentration of emetine in the lungs is ~173 and ~294 times higher than in plasma, respectively, with the maximum recorded amount present in the lungs at the 24-h time period. High concentrations were also found in many other tissues, such as the liver, heart, and small and large intestines [11]. Similarly, studies in humans have strongly indicated that emetine quickly undergoes extensive distribution to the tissues [9], with slow excretion and detectable concentrations that may persist in the urine for 40–60 days after treatment has been ceased [12].

It may therefore be possible that the lung concentrations of emetine after the oral administration of ipecac syrup are sufficient for anti-coronavirus activity, especially if other ipecac alkaloids in the syrup also have antiviral properties and it is taken with a 5-HT3 antagonist to avoid vomiting. As an example, cephaeline (another principal alkaloid of ipecac) has been shown to have low nanomolar IC_50_ (half-maximal inhibitory concentration) values of less than 0.042 μM against the Zika virus [13]. 

Unfortunately, emetine absorption from oral ipecac displays considerable inter-patient variation [14]. Some patients may not absorb sufficient emetine or alkaloids to be therapeutically effective. Intramuscular administration of emetine has a number of advantages over oral dosing. Emetine undergoes metabolism by the liver [15]. Intramuscular administration would prevent any liver metabolism during oral absorption (the “first-pass effect”). Radiolabeled emetine given parenterally, as with oral administration, accumulates in the lung [16]. In fact, another advantage of intramuscular over oral administration could be its greater uptake by the lungs. With radiolabeled emetine given by the oral route in rats, there was, at most time points, significantly (0.8–5.8 times) more emetine found in the liver than there was in the lungs [11]. Conversely, with radiolabeled emetine given by the intraperitoneal route in guinea pigs, the situation was reversed; at each time point, there was over twice as much emetine in the lungs than the liver [16]. It could initially be argued that this reflects interspecies variation. The more likely explanation is that with drugs absorbed into the intestinal circulation following oral administration, the first organ the circulation goes to is the liver, in order that foreign compounds can be detoxified. This results in a higher initial deposition in the liver, with the proportion in the liver decreasing throughout the day as it redistributes into the systemic circulation. With parenteral administration, once a drug enters the circulatory system, the first organ after leaving the heart is the lungs, resulting in higher concentrations to potentially counteract the COVID-19 virus.

Intramuscular administration of emetine can also result in nausea and vomiting [12] which, based on studies with ipecac syrup [10], are likely to be negated with the concomitant use of a 5-HT3 antagonist. It is unlikely that the two agents would be antagonistic to each other with regard to anti-viral activity, especially as emetine has a high affinity for the 5-HT4 receptor with little activity on 5-HT3 [17]. However, it should at least be considered that they could interact until in vitro studies can confirm otherwise.

But how can we be sure that effective concentrations are achieved by the intramuscular route? Emetine, given intramuscularly, has been established over a long period as the most specific and highly potent agent against intestinal and extra-intestinal amoebiasis [18]. The IC_50_ of emetine against *Entamoeba histolytica* is 26.8 μM [19]; this is approximately 500–1900 times higher than the EC_50_ for the SARS and MERS coronaviruses, implying that emetine is potentially far more potent as an anti-coronavirus agent than it is against amoebiasis. These results may cast doubt on the very low EC_50_ obtained with the SARS and MERS coronaviruses; however, studies by different authors have shown that emetine also has potent antiviral activity against the Zika virus (IC_50_ = 0.00874 μM) [13] and the human cytomegalovirus (EC_50_ = 0.04 μM) [20]. Interestingly, emetine also demonstrated a dose-dependent inhibition of Ebola virus viral-like particle entry into HeLa cells (IC_50_ = 10.2 μM) [13]. At 0.03 µM, emetine was able to reduce HIV (wild type and multi-drug resistant M184V) infection by up to 80% in peripheral blood mononuclear cells (PBMC) [21]. HIV reverse transcriptase was also reduced by approximately 50% at an emetine concentration of 10 μM [21]. In relation to the coronaviruses, emetine activity against four strains of coronavirus had EC_50_ values ranging from 0.12 to 1.43 μM, with the MERS coronavirus EC_50_ being 0.34 μM [22]. While this EC_50_ for the MERS virus is higher than in previous studies [3], it does indicate that emetine is highly active against multiple coronaviruses. It has also been demonstrated that emetine can reduce viral entry into DPP4-expressing Huh-7.5 cells by a factor of 50-fold compared with that of the control, with an EC_50_ value of 0.16 μM [22].

The main therapeutic issue with emetine use is perhaps its potential for cardiac toxicity. This was especially prevalent in India, where it was estimated that 10%–40% of the population had suffered from amoebiasis, resulting in the use of emetine being “widespread and lavish” [18]. Emetine use was associated with changes in the electrocardiogram (ECG) —in particular, prolongation of the QT interval, elevation of the ST segment and inversion of the T wave [18]. A review in 1980 of the cardiac toxicity found that at therapeutic doses (1 mg/kg intramuscularly, maximum 60 mg, per day for 10 days or less [5]) non-permanent cardiovascular side effects occurred. This frequently included ECG changes and moderate hypotension [5] and occasionally tachycardia and precordial pain. These changes occurred during treatment or after completion of treatment and often lasted for a period of time. The patient usually recovered without any sustained change in cardiovascular function [5]. Similarly, chronic ingestion of ipecac syrup over many months by sufferers of bulimia nervosa has been associated with cardiac fractional shortening due to cardiomyopathy, but this has been known to revert to normal after the ipecac was ceased [23]. While rare, deaths from ipecac syrup overuse have been reported [24]. 

Considering the substantially higher potency that emetine appears to have against the coronavirus, doses of one fifth to one tenth of the doses used for the treatment of amoebiasis (0.1–0.2 mg/kg, intramuscularly; maximum 6-12 mg/day) could potentially be used. These lower doses are likely to minimise or eliminate any significant cardiac toxicity and nausea while maintaining antiviral effectiveness. It should also be noted that the intravenous route was considered too toxic and offered no therapeutic advantages [6], and appropriate pharmaceutical references such as those listed [6,12] should be consulted before clinical use in patients. For the formulation and testing of an emetine injection, both the United States Pharmacopoeia and the British Pharmacopoeia (BP) have listed Emetine Injections [12], with emetine only being omitted from the BP 2013 edition onwards [25], and still available as a reference standard from the US Pharmacopoeia website [26].

If it was used in patients, it is beyond the scope of this article to suggest at which stage in the disease process an agent such as emetine should be used to treat coronavirus infection. Too early and prolific use could promote resistance. If therapy is left too late when acute respiratory distress [27] has developed, it may limit the effectiveness of the drug.

A related compound to emetine that should be tested in vitro for coronavirus sensitivity is dehydroemetine, which was developed in response to the cardiovascular toxicity associated with emetine. Dehydroemetine is structurally similar to emetine but is recognised as having a lower cardiovascular risk profile then emetine [18] and has been used as a replacement for many years. Dehydroemetine is eliminated from tissues more quickly [16], which may explain its lower toxicity. However, at present, there are little sensitivity data for the use of dehydroemetine as an antiviral agent.

Under normal situations, it would be unexpected that an antibacterial compound would also be an antiviral compound. More unlikely, that the same mode of action would account for both antibacterial and antiviral activities. As emetine affects ribosomal protein synthesis in yeast and has been shown to inhibit viral-RNA synthesis [28], there may be some overlap between its antibacterial and antiviral modes of action. If this is the case, the significant structural activity relationships gained from the emetine analogues [28] could be applied to the development of new antiviral agents.

Emetine was selected from a list of drugs that had been tested in vitro for their antiviral activity against coronaviruses [2]; see Table 1. For each drug, a mid-range achievable blood level was obtained from the literature. This blood level was, in part, arbitrary as blood levels are mostly determined by the dose, which can vary considerably based on the condition being treated; there is also limited availability of studies measuring blood concentrations in patients. The ratio of the blood concentration/IC_50_ was used to initially rank a drug’s potential clinical significance as an antiviral agent against coronaviruses. Drugs that had higher ratios were deemed to be more clinically important, as it signified that therapeutic antiviral concentrations were more likely to be achievable at normal dosing. This system, by itself, proved inadequate, especially in the case of emetine. First, blood levels varied between studies. One study reported the range [14] of levels achieved, while another recorded the mean [9], with the midpoint of the range and the mean being very different. This initially indicated that emetine could be very useful, with a blood concentration/IC_50_ ratio of approximately 8 (see Table 1). When a more realistic blood concentration using the mean was found, the ratio was less than 2. Second, the blood levels do not take into consideration the tissue distribution of the drug. Basic pharmacokinetic parameters of each drug, such as the volume of distribution as an indicator of tissue uptake, should have been used to identify drugs that could potentially exhibit favourable tissue distribution. However, the volume of distribution data alone is not sufficient to determine specific tissue accumulation. A high volume of distribution indicates that the drug is removed from the plasma and goes into the tissues, but which tissues? Lipophilic drugs, with a high volume of distribution, are of little use if the drug partitions solely into adipose tissue. Similarly, a high distribution into the bones, as for bisphosphonates [29], would also be of little use in this scenario. Emetine has good radiolabeled studies [11,16] that clearly showed that not only was the bulk of the emetine distributed in the tissues, but also a significant portion went to the lungs.

Regardless, Table 1 is still able to highlight some drugs that may be of use. This would include agents such as lopinavir, hydroxychloroquine and mycophenolate with or without interferon beta-1b. In the case of mycophenolate with interferon beta-1b, it appears to have been therapeutically effective in treating patients with MERS [30]. Given the blood concentration/IC_50_ ratio of ~30, calculated on trough levels for mycophenolate with interferon beta-1b, sub-therapeutic immunosuppressive doses may still have antiviral efficacy, especially if used in combination with other drugs such as hydroxychloroquine or lopinavir. From animal studies, interferon beta-1b appears to exhibit antiviral activity against the coronavirus on its own [31].

From Table 2, adapted from Salata 2017 [32], it can be seen that commercially available cationic amphiphilic drugs screened for their antiviral activity often exhibit antiviral activity against different types or families of viruses, with similar IC_50_/EC_50_. It is possible that a number of agents from this list could also be active against the COVID-19 virus. 

At present, emetine has advantages which, when considered together, make it an attractive candidate: it significantly inhibits two coronaviruses in the low nano-molar range; it potentially achieves satisfactory plasma levels when administered orally; and it produces substantially higher concentrations in affected tissues (the lungs) that are known to effectively treat *Entamoeba histolytica* infections, which have inhibitory concentrations hundreds of times higher than at least two coronaviruses. Emetine also has a long and broad history of use, and its side effects are well known and are manageable.

Given the serious and immediate threat that the COVID-19 virus poses, our long if not partially forgotten history with emetine and dehydroemetine, and the likely ability of emetine to reach therapeutic concentrations within the lungs, ipecac, emetine, and other analogues should be considered as potential treatment options, especially if in vitro studies confirm that the COVID-19 virus is sensitive to these agents.

**Table 1:** Inhibitory activities of the reported MERS-CoV replication inhibitors available in the FDA-approved drug register (adapted from Liu 2015 [2]) with approximate therapeutic blood concentrations and corresponding ratio of blood concentration and IC_50_.

	IC_50_ μM (a)	MW g/mole	Blood [Conc. μM] (b)	Ratio: (Blood [Conc μM])/IC_50_	Blood [Conc.] Ref.	Additional Notes
Lopinavir	17	628.81	11.45	0.67	[33]	Cmin 7.2 mg/L, 400 mg twice daily
Loperamide	5.9	477	0.001	<0.005	[34]	~0.5 ng/mL after 4 mg dose
Chloroquine	5.2	319.9	1.56	0.30	[35]	~0.5 mg/L dose, 450 mg/day
Hydroxychloroquine	8.28	335.9	3.72	0.45	[36]	range of 500–2000 ng/mL, dose maximum of 200–400 mg/day depending on renal function
Amodiaquine	6.21	355.86	0.76	0.12	[37]	~270 ng/mL, 10 mg/kg single dose; Is rapidly metabolised to desethylamodiaquine – blood level is for metabolite (assumed to be as active as parent)
Chlorpromazine	9.15	318.86	0.52	0.06	[38]	Reference range: 30–300 ng/mL Large volume of distribution [38]
Promethazine	11.8	284.4	0.02	<0.005	[39]	~5 ng/mL peak, 25 mg oral single dose Large volume of distribution [39]
Fluphenazine	5.86	437.52	0.01	<0.005	[38]	Reference range: 1–10 ng/mL Large volume of distribution [38]
Thiothixene	9.3	443.6	0.05	<0.005	[40]	3–45 ng/mL, 20 mg dose
Astemizole	4.88	458.57	0.17	0.04	[41]	~70 μg/L peak, 300 mg single oral dose. Blood level was astemizole + hydroxylated (active) metabolites. Large volume of distribution.
Triflupromazine	5.76	352.418				Insufficient data to make assessment
Clomipramine	9.33	314.9	1.05	0.11	[38]	Reference range: 230–450 ng/mL Large volume of distribution [38]
Emetine	0.01	480.64	0.08	8.32	[14]	5 to 73 ng/mL, 11.4 mg oral dose. The blood concentration used was the midpoint in subjects with detectable levels of emetine
Tamoxifen	10.12	371.5	0.32	0.03	[42]	120 ng/mL, 20 mg daily
Cycloheximide	0.19					Too toxic [43]
Dasatinib	5.47	488.01	0.003	<0.005	[44]	Cmin to <3 nmol/l
Ribavirin (c)	40.9	244.2	8.19	0.20	[45]	<30% of patients obtained this concentration 2 mg/l after 24 weeks [45]
Mycophenolic acid (c) (d)	0.53	320.34	6.24	11.78	[46]	The blood concentrations are the troughs; the peaks are substantially higher. Calculated on a trough of 2 μg/mL
Mycophenolic acid + 12.5 IU/mL interferon beta-1b (d) (e)	0.187	320.34	6.24	33.39	[46,47]	The blood concentrations are the troughs; the peaks are substantially higher. Calculated on a trough of 2 μg/mL

Notes: Data from Liu 2015 [2] unless otherwise stated. (b) Blood levels cannot be exact and will vary depending on dose, condition being treated and available studies. (c) Value calculated from Chan 2013 [48] (d) Mycophenolic acid is the active metabolite of mycophenolate. Mycophenolate with interferon beta-1b appears to have been therapeutically effective in treating patients with MERS [30].

**Table 2:** Antiviral efficacy of selected cationic amphiphilic drugs, adapted from Salata 2017 [32].

Drug	Antiviral Efficacy
Amiodarone	Filovirus—IC_50_ 0.25–1.38 μg/mL Ebola virus—IC_50_ 5.60 μM HCV—EC_50_ 2.10 μM
Bepridil	Ebola virus—IC_50_ 3.21–5.08 μM
Chloroquine and Hydroxychloroquine	CCHFV—IC_50_ 28.00–43.00 μM Filovirus—EC_50_ 4.70–15.00 μM HCoV-OC43—EC_50_ 0.306 μM KSHV—IC_50_ 3.30–5.10 μM MERS-CoV—EC_50_ 3.00–6.28 μM SARS-CoV—EC_50_ 6.54–8.80 μM Dengue virus type 2—EC_50_ 9.70–12.90 μM KSHV—IC_50_ 1.30 μM
Quinacrine	Dengue virus type 2—EC_50_ 7.09 μM Zika virus—EC_50_ 2.27 μM
Mefloquine	Dengue virus type 2 – EC_50_ 4.36 μM Zika virus—EC_50_ 3.95 μM
Chlorpromazine	CCHFV—IC_50_ 10.80–15.70 μM MERS-CoV—EC_50_ 4.90–9.51 μM SARS-CoV—EC_50_ 12.97 μM
Promethazine	Filovirus—IC_50_ 19.10–19.40 μM
Sertraline	Ebola virus—IC_50_ 1.44–3.13 μM
Trimipramine	Filovirus—IC_50_ 10.90–11.10 μM
Clomiphene	Filovirus—IC_50_ 0.76–11.10 μM HCV—EC_50_
Tamoxifen	HCV—EC_50_ 0.10 μM HSV—IC_50_ 4.89 μM MERS-CoV—EC_50_ 10.12 μM SARS-CoV—EC_50_ 92.89 μM
Toremifene	Filovirus—IC_50_ 0.03–6.17 μM MERS-CoV—EC_50_ 12.92 μM SARS-CoV—EC_50_ 11.97 μM
Sunitinib	HCV—IC_50_ 0.05 μM
Terconazole	Ebola virus—IC_50_ 7.07–8.26 μM
Triparanol	Ebola virus—IC_50_ 1.92 μM
